# Knockdown of TUG1 rescues cardiomyocyte hypertrophy through targeting the miR-497/MEF2C axis

**DOI:** 10.1515/biol-2021-0025

**Published:** 2021-03-16

**Authors:** Guorong Zhang, Xinghua Ni

**Affiliations:** Department of Internal Medicine-Cardiovascular, The Fourth Affiliated Hospital of Nanchang University, No. 133 The South Guangchang Road, Nanchang 330003, Jiangxi, China; Department of the Seventh Medical Center, PLA General Hospital, Beijing, China

**Keywords:** cardiac hypertrophy, TUG1, miR-497, myocyte enhancer factor 2C

## Abstract

The aim of this study was to investigate the detailed role and molecular mechanism of long noncoding RNA (lncRNA) taurine upregulated gene 1 (TUG1) in cardiac hypertrophy. Cardiac hypertrophy was established by transverse abdominal aortic constriction (TAC) *in vivo* or angiotensin II (Ang II) treatment *in vitro*. Levels of lncRNA TUG1, miR-497 and myocyte enhancer factor 2C (MEF2C) mRNA were assessed by quantitative reverse transcriptase PCR (qRT-PCR). Western blot assay was performed to determine the expression of MEF2C protein. The endogenous interactions among TUG1, miR-497 and MEF2C were confirmed by dual-luciferase reporter and RNA immunoprecipitation assays. Our data indicated that TUG1 was upregulated and miR-497 was downregulated in the TAC rat model and Ang II-induced cardiomyocytes. TUG1 knockdown or miR-497 overexpression alleviated the hypertrophy induced by Ang II in cardiomyocytes. Moreover, TUG1 acted as a sponge of miR-497, and MEF2C was directly targeted and repressed by miR-497. miR-497 overexpression mediated the protective role of TUG1 knockdown in Ang II-induced cardiomyocyte hypertrophy. MEF2C was a functional target of miR-497 in regulating Ang II-induced cardiomyocyte hypertrophy. In addition, TUG1 regulated MEF2C expression through sponging miR-497. Knockdown of TUG1 rescued Ang II-induced hypertrophy in cardiomyocytes at least partly through targeting the miR-497/MEF2C axis, highlighting a novel promising therapeutic target for cardiac hypertrophy treatment.

## 1 Introduction

Cardiac hypertrophy is a common physiological compensatory response of the heart against a number of stressors to maintain normal cardiac function. However, enlargement of the heart in response to myocardial injury, hypertensive stress or excessive neurohumoral activation is associated with maladaptive remodeling and cardiac dysfunction and is classified as pathological hypertrophy [1]. Pathological cardiac hypertrophy is a major risk factor for diomyopathy, heart failure and sudden cardiac death [2,3]. Despite the improvements of the understanding of pathological regulators in cardiac hypertrophy [4,5], the molecular mechanisms of cardiac hypertrophy remain largely unclear.

Long noncoding RNAs (lncRNAs) are more than 200 nucleotide RNA molecules that perform various functions in a series of important biological processes [6]. They have been discovered to have relevance to human diseases, including cardiac hypertrophy [7]. For example, Wang et al. reported that cardiac hypertrophy-related factor (CHRF) regulated cardiac hypertrophy by acting as a sponge of microRNA (miRNA)-489 [8]. Wang et al. identified that cardiac hypertrophy-associated epigenetic regulator (Chaer) worked as an epigenetic checkpoint in cardiac hypertrophy [9]. A recent study demonstrated that *taurine upregulated gene 1* (*TUG1*) was involved in the pathogenesis of cardiac hypertrophy by sponging miRNA-29b-3p [10]. Herein, we identified the functional role and the underlying mechanisms of TUG1 in cardiac hypertrophy.

miRNAs, a class of endogenous, small noncoding RNAs with 20–22 nucleotides, are known to be present in the RNA-induced silencing complexes (RISCs), where they silence gene expression [11]. Dysregulation of miRNAs is found to have relevance to human diseases, including cardiac hypertrophy [12]. miR-497, a member of the miR-15 family, was identified as a novel regulator of cardiac hypertrophy and fibrosis by the repression of the TGFβ-pathway [13]. A recent study demonstrated that the increased expression of miR-497 ameliorated cardiac hypertrophy *in vitro* and *in vivo* via targeting sirtuin 4 (Sirt4) [14]. Previous studies had reported that TUG1 exerted a regulatory function in human disease through the competing endogenous RNA (ceRNA) network via sponging miRNAs [15,16]. However, the effect of interplay between TUG1 and miR-497 in cardiac hypertrophy remains undefined. In the present study, our data supported that TUG1 was upregulated in the TAC rat model and angiotensin II (Ang II)-induced cardiomyocytes. Furthermore, TUG1 knockdown attenuated cardiomyocyte hypertrophy *in vitro* by targeting the miR-497/myocyte enhancer factor 2C (MEF2C) axis.

## 2 Materials and methods

### 2.1 Animal care and use

Female Sprague-Dawley (SD) rats (8 weeks old, 190–220 g) were purchased from Henan Research Center of Laboratory Animal (Zhengzhou, Henan, China) and housed in a specific pathogen-free environment in the animal facility of the Fourth Affiliated Hospital of Nanchang University. All rats were kept at a constant temperature (22 ± 2°C) with 60% humidity and a 12 h light–dark cycle and fed a standard chow diet for at least 1 week before experimentation. All animal experimental procedures were performed in accordance with the Agriculture Guidebook of the Care and Use of Laboratory Animals.


**Ethical approval:** The research related to animal use has been complied with all the relevant national regulations and institutional policies for the care and use of animals and was approved by the Animal Ethics Committee of the Fourth Affiliated Hospital of Nanchang University.

### 2.2 Animal model

Experimental rats were divided into two groups: (1) Sham group (*n* = 8), in which rats underwent exposure of abdominal aorta without ligation, and (2) transverse abdominal aortic constriction (TAC) group (*n* = 8), in which abdominal aorta of rats was exposed and ligated. The construct of the TAC rat model was performed as described previously [17]. Briefly, a 1.5–2.0 cm longitudinal incision was exposed to the left side of the rat abdomen. Then, a 4-0 suture was used to tie two circles around the abdominal aorta by a 12-gauge needle, after which the needle was eliminated. At 8 weeks after surgery, cardiac dimensions and function were analyzed using a Doppler echocardiography (Philios IE33 ultrasound system, Phillips Medical Systems, Andover, MA, USA) including the left ventricular end-diastolic dimension (LVEDD), the left ventricular end-systolic diameter (LVESD), the left ventricular end-systolic pressure (LVESP), the left ventricular end-diastolic pressure (LVEDP) and fractional shortening (FS). Subsequently, all rats were euthanized; the ratios of heart weight and body weight were determined; and heart tissues were collected for further evaluation.

### 2.3 Primary cardiomyocyte isolation, culture and treatment

Primary cardiomyocytes were isolated from the hearts of 1- to 3-day-old newborn SD rats as described previously with modification [18]. In brief, newborn rats were euthanized, and the hearts were immediately excised and minced. The heart tissues were digested with 0.1% collagenase type II (Gibco, Rockville, MD, USA) and 0.1% trypsin (Gibco) at 37°C. After centrifugation, cardiomyocytes were harvested and maintained in Dulbecco’s Modified Eagle Medium/Nutrient Mixture F-12 (DMEM/F-12, Gibco) supplemented with 10% fetal bovine serum (FBS, Gibco) and 1% penicillin/streptomycin (Gibco) in an incubator at 37°C with 5% CO_2_. To establish a cardiomyocyte hypertrophy model *in vitro*, the cardiomyocytes of ∼70% were treated with 1 mM of Ang II (Sigma-Aldrich, St. Louis, MO, USA) for 48 h.

### 2.4 Cell transfection

For TUG1 knockdown studies, cardiomyocytes were transiently introduced with siRNA targeting TUG1 (si-TUG1, GenePharma, Shanghai, China) or a nontarget siRNA (si-NC, GenePharma). For miR-497 overexpression, cardiomyocytes were transfected with the mature miR-497 mimic (GenePharma) or a scrambled oligonucleotide sequence (miR-NC mimic, GenePharma). miR-497 silencing was carried out using miR-497 inhibitor (in-miR-497, GenePharma), and in-miR-NC (GenePharma) was used as a negative control. For overexpression experiments, cardiomyocytes were introduced with pcDNA-based TUG1 or a MEF2C overexpression plasmid (pcDNA-TUG1 or pcDNA-MEF2C, GenePharma) or a negative control plasmid (pcDNA-NC, GenePharma). All transfections were performed using Lipofectamine 2000 transfection reagent (Invitrogen, Waltham, MA, USA) following the protocols of manufacturers.

### 2.5 Quantitative reverse transcriptase PCR

Total RNA was extracted from heart tissues and cardiomyocytes using RNA Purification kit (Invitrogen) in accordance with manufacturer’s instructions. The quality and quantity of RNA extracts were assessed by a NanoDrop ND-2000 Spectrophotometer (NanoDrop Technologies, Wilmington, DE, USA). The levels of atrial natriuretic peptide (ANP), brain natriuretic peptide (BNP), β-myosin heavy chain (β-MHC), TUG1 and MEF2C mRNA were detected by using quantitative reverse transcriptase PCR (qRT-PCR). Total RNA (1 µg) was reverse transcribed into cDNA using M-MLV reverse transcriptase (Invitrogen), and qRT-PCR was carried out using PowerUp^TM^ SYBR^TM^ Green PCR Master Mix (Applied Biosystems) on the ABI 7900HT sequence detector (Applied Biosystems) following the protocols of manufacturers. The indicated gene expression levels were normalized against glyceraldehyde-3-phosphate dehydrogenase (GAPDH). The level of mature miR-497 was determined by qRT-PCR using TaqMan reverse transcription kit (Applied Biosystems, Foster City, CA, USA) and TaqMan MicroRNA assay kit (Applied Biosystems) with snRNA RNU6B as an internal control. The amplification profile was denatured at 95°C for 10 min, followed by 40 cycles of 95°C for 20 s and 60°C for 1 min. Relative gene expression was calculated based on the 2^−∆∆Ct^ method.

### 2.6 Western blot

Total protein was prepared in ice-cold RIPA buffer (150 mM Tris-HCl, pH = 7.6, 150 mM NaCl, 0.5% Triton X-100, 0.1% SDS, 1 mM phenylmethanesulfonyl fluoride, 1 mM Na_3_VO_4_) containing a cocktail of protease inhibitors (Thermo Fisher Scientific, Waltham, MA, USA) and then quantified using a BCA Protein assay kit (Thermo Fisher Scientific). Protein extracts were resolved on a 10% SDS-polyacrylamide gel and electrophoretically transferred onto a polyvinylidene fluoride (PVDF) membrane (Millipore, Bedford, MA, USA). The following primary antibodies were used: anti-ANP (Abcam, Cambridge, UK; dilution 1:1,000), anti-BNP (Abcam; dilution 1:500), anti-β-MHC (Abcam; dilution 1:1,000), anti-MEF2C (Abcam; dilution 1:1,000) and anti-β-actin (Abcam; dilution 1:3,000). The horseradish peroxidase-conjugated anti-rabbit (Abcam; dilution 1:5,000) or anti-mouse (Abcam; dilution 1:5,000) IgG was used as a secondary antibody. Protein bands were detected using the enhanced chemiluminescence (ECL) detection kit (Immobilon Western Chemiluminescent HRP Substrate, Millipore) and analyzed using ImageJ software (National Institutes of Health, Bethesda, MD, USA).

### 2.7 Bioinformatic analysis and dual-luciferase reporter assay

Online database LncBase Predicted v.2 (http://carolina.imis.athena-innovation.gr/diana_tools/web/index.php?r=lncbasev2%2Findex-predicted) was used to help identify the miRNAs that potentially bind to TUG1. Bioinformatic analysis for the molecular targets of miR-497 was performed using microT-CDS software at http://diana.imis.athena-innovation.gr/DianaTools/index.php?r=microT_CDS/index. TUG1 and MEF2C 3′UTR luciferase reporter plasmids (TUG1-WT and MEF2C 3′UTR-WT) harboring the miR-497-binding sites and the site-directed mutations of the seed region (TUG1-MUT and MEF2C 3′UTR-MUT) were obtained from GenePharma. The cardiomyocytes were cotransfected with each reporter construct and miR-NC mimic or miR-497 mimic. After 48 h transfection, the luciferase activity was determined using a dual-luciferase reporter assay system (Promege, Madison, WI, USA) following manufacturer’s guidance.

### 2.8 RNA immunoprecipitation assay

RNA immunoprecipitation (RIP) assay was carried out using the Magna RIP RNA Immunoprecipitation kit (Millipore) with anti-Argonaute 2 (anti-Ago2, Abcam) antibody. Briefly, cell lysates were prepared using the ice-cold RIPA buffer and then incubated with anti-Ago2 or negative control IgG antibody at 4°C for 4 h, followed by the incubation with protein A/G agarose for 4 h. Beads were harvested by centrifugation and washed three times using ice-cold PBS. Next, total RNA was extracted, and the enrichment levels of TUG1 and MEF2C mRNA were detected by qRT-PCR.

### 2.9 Statistical analysis

All data were analyzed using GraphPad Prism 5.0 software (GraphPad Software, Inc., San Diego, CA, USA) and expressed as mean ± standard deviation (SD). All experiments were carried out in triplicate. Differences between groups were compared by Student’s *t*-test or analysis of variance (ANOVA). A probability value of *P* < 0.05 was considered to be significant.

## 3 Results

### 3.1 TUG1 level was upregulated in the TAC rat model and Ang II-induced cardiomyocytes

**Figure 1:**
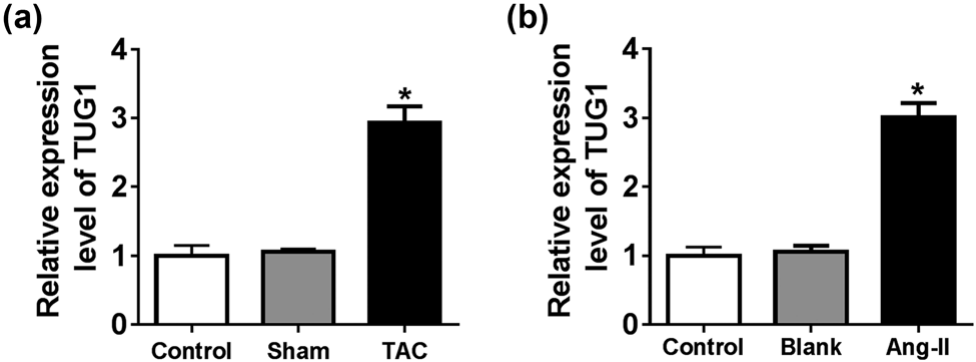
TUG1 was upregulated in the TAC rat model and Ang II-treated cardiomyocytes. Relative TUG1 level by qRT-PCR in the untreated heart tissues (control), Sham model and TAC model (a), and untreated (Control), Mock-treated (Blank) and Ang II-treated cardiomyocytes (b). *P < 0.05.

**Figure A1:**
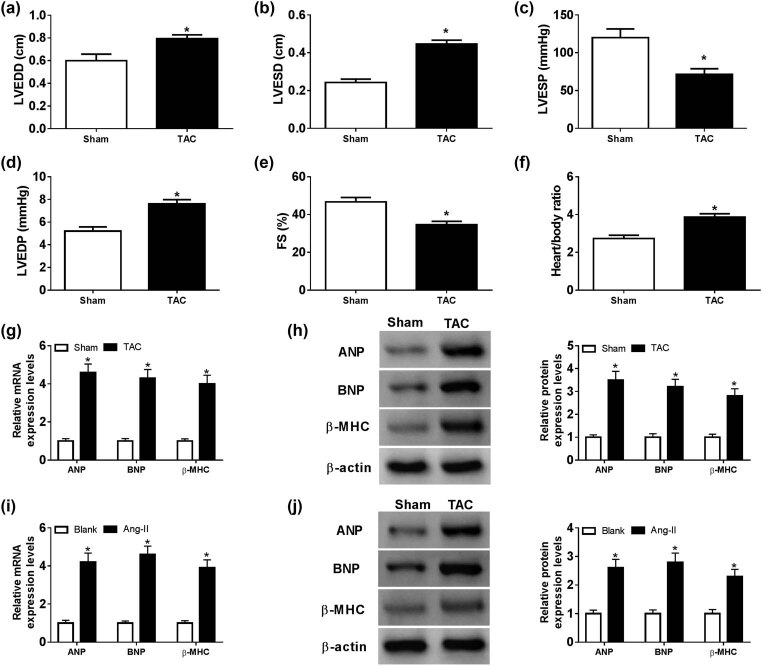
The establishment of cardiac hypertrophy model by TAC *in vivo* and Ang II treatment *in vitro*. Analysis for LVEDD (a), LVESD (b), LVESP (c), LVEDP (d) and FS (e), heart weight/body weight ratio (f) in the TAC rat model. mRNA levels of ANP, BNP and β-MHC by qRT-PCR (g), and their protein levels by western blot (h) in the TAC rat model. Primary cardiomyocytes were treated with 1 mM of Ang II for 48 h, followed by the detection of ANP, BNP and β-MHC mRNA levels by qRT-PCR (i) and their protein levels by western blot (j). *P < 0.05.

To investigate the involvement of TUG1 in cardiac hypertrophy, we first established the cardiac hypertrophy model by TAC *in vivo* and Ang II treatment *in vitro*. By contrast, TAC led to a significant increase of LVEDD, LVESD and LVEDP and a striking reduction of LVESP and FS, as well as a strong elevation of the radio of heart weight and body weight (Figure A1a–f). Moreover, TAC caused a remarkable increase in the levels of hypertrophy-related genes ANP, BNP and β-MHC (Figure A1g and h), demonstrating the successful establishment for the TAC rat model. Furthermore, Ang II treatment remarkably increased the levels of ANP, BNP and β-MHC in cardiomyocytes (Figure A1i and j). Interestingly, as shown by qRT-PCR, the TUG1 level was significantly upregulated in the TAC model and Ang II-treated cardiomyocytes (Figure 1a and b).

### 3.2 Knockdown of TUG1 attenuated Ang II-induced cardiomyocyte hypertrophy

**Figure 2:**
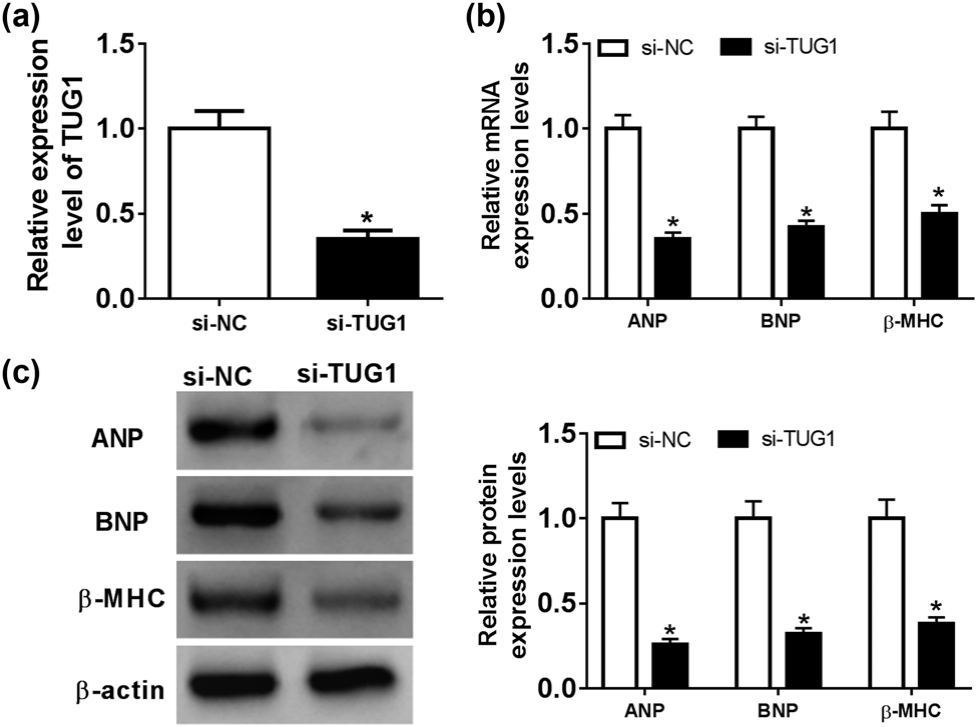
TUG1 knockdown attenuated Ang II-induced cardiomyocyte hypertrophy. Cardiomyocytes were transfected with si-NC or si-TUG1 for 24 h and then treated with 1 mM of Ang II for 48 h, followed by the determination of TUG1 expression by qRT-PCR (a), the mRNA levels of ANP, BNP and β-MHC by qRT-PCR (b) and their protein levels by western blot (c). *P < 0.05.

To further investigate the functional role of TUG1 in cardiac hypertrophy, we performed loss-of-function experiments with si-TUG1. Transient transfection of si-TUG1, but not a scrambled control sequence, resulted in a significant decrease in the level of TUG1 in Ang II-treated cardiomyocytes (Figure 2a). Subsequent qRT-PCR and western blot assays revealed that in comparison to the negative control, TUG1 knockdown triggered a remarkable reduction in the expression of ANP, BNP and β-MHC at both mRNA and protein levels in Ang II-treated cardiomyocytes (Figure 2b and c).

### 3.3 TUG1 acted as a molecular sponge of miR-497

**Figure 3:**
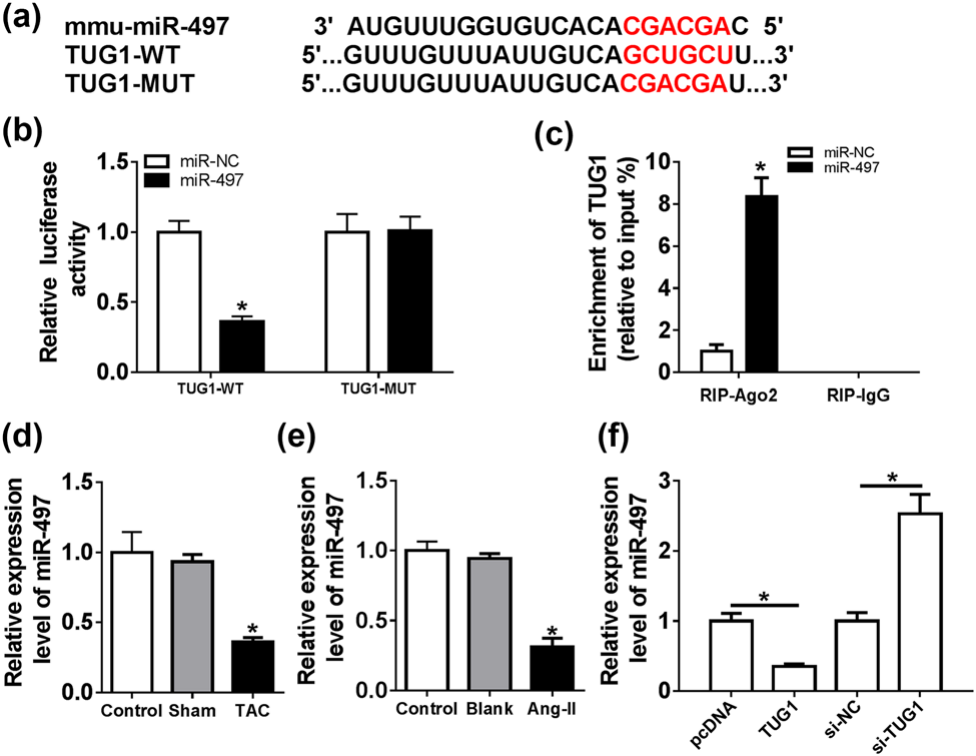
TUG1 acted as a molecular sponge of miR-497. (a) Schematic of the miR-497-binding sites within TUG1 and mutated miR-497-binding sites. (b) Relative luciferase activity in cardiomyocytes cotransfected with TUG1-WT or TUG1-MUT and miR-NC mimic or miR-497 mimic. (c) TUG1 enrichment in the RISC of cardiomyocytes transfected with miR-NC mimic or miR-497 mimic using anti-Ago2 antibody. TUG1 expression by qRT-PCR in the untreated heart tissues (control), Sham model and TAC model (d), and untreated (control), Mock-treated (Blank) and Ang II-induced cardiomyocytes (e) and cardiomyocytes transfected with pcDNA, pcDNA-TUG1, si-NC or si-TUG1 (f). *P < 0.05.

**Figure A2:**
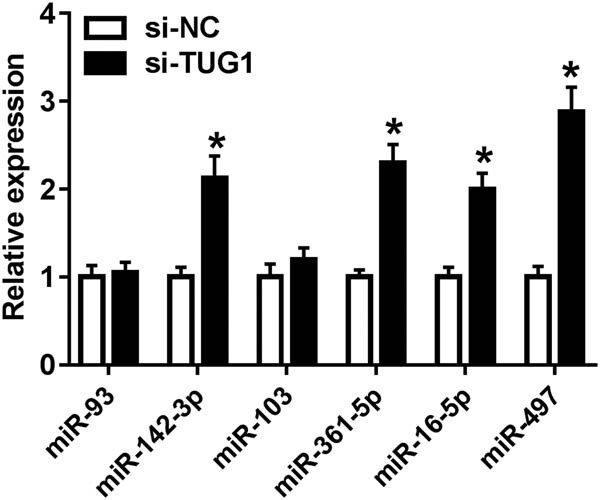
The effect of TUG1 on the expression of six miRNAs in cardiomyocytes. Relative expression levels of miR-93, miR-142-3p, miR-103, miR-631-5p, miR-16-5p and miR-497 by qRT-PCR in cardiomyocytes transfected with si-NC or si-TUG1. *P < 0.05.

To further understand the function of TUG1 in cardiac hypertrophy, we used online database LncBase Predicted v.2 to help identify the miRNAs that potentially bind to TUG1. Among these candidates, we selected several miRNAs (miR-93, miR-142-3p, miR-103, miR-631-5p, miR-16-5p and miR-497), which were related to cardiomyocyte hypertrophy and were downregulated in cardiomyocyte hypertrophy. Our results showed that miR-497 was the most significantly upregulated miRNA in TUG1-silencing cardiomyocytes (Figure A2), and thus, we selected miR-497 for further investigation. These data revealed a putative target sequence for miR-497 within TUG1 (Figure 3a). To validate this, we carried out dual-luciferase reporter assays. When we cloned the TUG1 segment harboring the miR-497-binding sequence into a luciferase reporter, the cotransfection of TUG1 wild-type reporter and miR-497 mimic into cardiomyocytes caused lower luciferase activity than cells cotransfected with miR-NC mimic (Figure 3b). However, when the target sites were mutated, no reduction was observed in luciferase in the presence of miR-497 mimic (Figure 3b). Ago2, a core component of the RISC, acts as a crucial regulator in the mature process of miRNA [11]. Thus, RIP experiments were done using anti-Ago2 antibody. The data showed that TUG1 enrichment was significantly elevated by miR-497 overexpression (Figure 3c), eliciting the potential endogenous interaction between TUG1 and miR-497. The data of qRT-PCR also revealed that miR-497 was prominently downregulated in the TAC model and Ang II-treated cardiomyocytes (Figure 3d and e). Furthermore, in comparison to their counterparts, miR-497 expression was significantly decreased by TUG1 overexpression plasmid, and it was remarkably increased in case of TUG1 depletion in cardiomyocytes (Figure 3f).

### 3.4 miR-497 overexpression mediated the protective role of TUG1 knockdown in Ang II-induced cardiomyocyte hypertrophy

**Figure 4:**
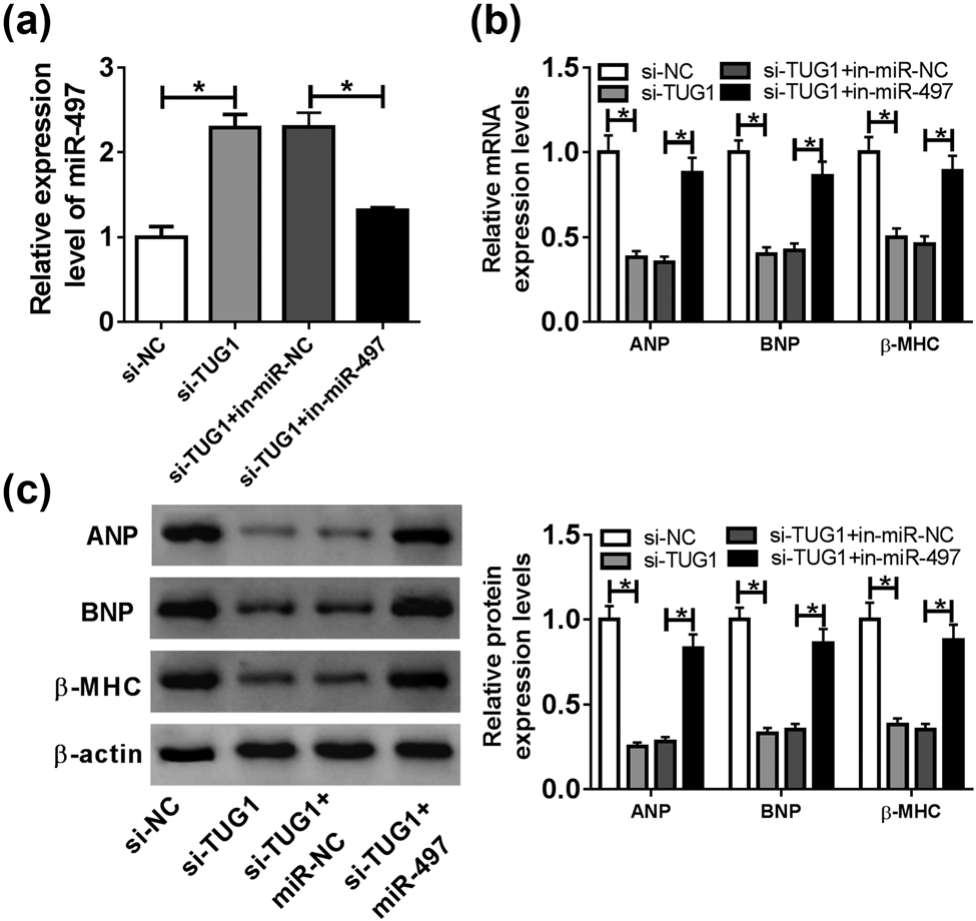
miR-497 overexpression mediated the protective role of TUG1 knockdown in Ang II-induced cardiomyocyte hypertrophy. Cardiomyocytes were transfected with si-NC, si-TUG1, si-TUG1 + in-miR-NC or si-TUG1 + in-miR-497 before Ang II exposure, followed by the measurement of miR-497 expression by qRT-PCR (a), the mRNA levels of ANP, BNP and β-MHC by qRT-PCR (b) and their protein levels by western blot (c). *P < 0.05.

To determine whether TUG1 modulated cardiomyocyte hypertrophy by miR-497, we reduced miR-497 expression in si-TUC1-transfected cardiomyocytes before Ang II treatment. The results of qRT-PCR showed that si-TUG1-mediated miR-497 upregulation was strikingly reversed by in-miR-497 transfection (Figure 4a). Moreover, the reduced effects of TUG1 knockdown on ANP, BNP and β-MHC expression were significantly abolished by miR-497 level restoration in Ang II-treated cardiomyocytes (Figure 4b and c).

### 3.5 TUG1-regulated MEF2C expression through sponging miR-497

**Figure 5:**
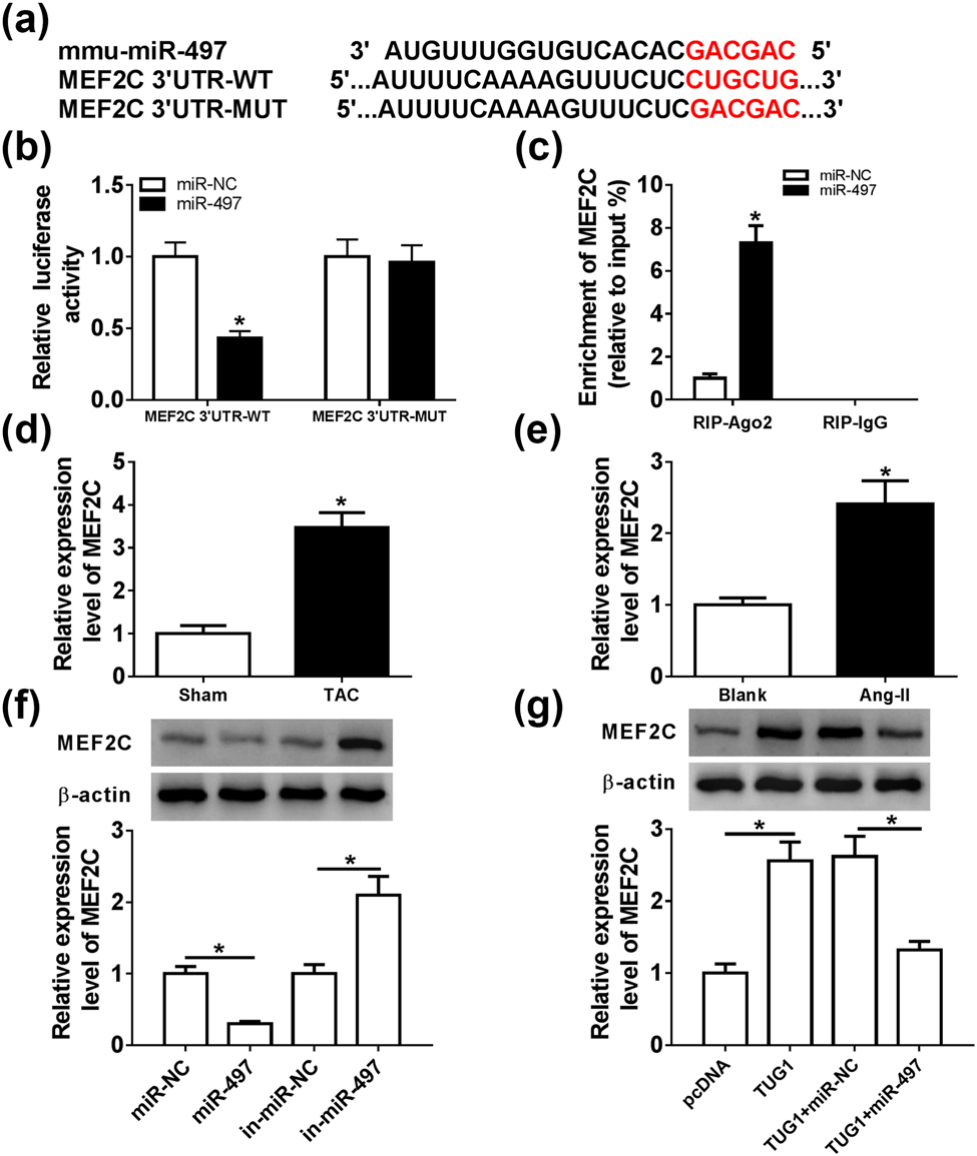
TUG1-regulated MEF2C expression through sponging miR-497. (a) Nucleotide resolution of the predicted miR-497-binding sites within the 3′UTR of MEF2C mRNA and the mutant of the seed region. (b) Relative luciferase activity in cardiomyocytes cotransfected with MEF2C 3′UTR-WT or MEF2C 3′UTR-MUT and miR-NC mimic or miR-497 mimic. (c) The enrichment of MEF2C mRNA in the RISC of cardiomyocytes transfected with miR-NC mimic or miR-497 mimic using anti-Ago2 antibody. MEF2C expression by qRT-PCR or western blot in TAC model (d), Ang II-treated cardiomyocytes (e) and cardiomyocytes transfected with miR-NC mimic, miR-497 mimic, in-miR-NC or in-miR-497 (f). (g) MEF2C expression by western blot in cardiomyocytes transfected with pcDNA, pcDNA-TUG1, pcDNA-TUG1 + miR-NC mimic or pcDNA-TUG1 + miR-497 mimic. *P < 0.05.

miRNAs exert biological function by regulating their target genes. Herein, to further understand the underlying mechanism by which miR-497 regulated cardiac hypertrophy, we carried out a detailed analysis for its molecular targets. Using microT-CDS software, the predicted data showed a putative complementary sequence for miR-497 within the 3′UTR of MEF2C mRNA (Figure 5a). To confirm whether MEF2C was a direct target of miR-497, dual-luciferase reporter assays were performed using an MEF2C 3′UTR reporter (MEF2C 3′UTR-WT) harboring the miR-497-binding sequence and the site-directed mutation of the seed region (MEF2C 3′UTR-MUT). In contrast to the negative control, the transfection of miR-497 mimic significantly reduced the luciferase activity of MEF2C 3′UTR-WT (Figure 5b). However, the site-directed mutation of the miR-497-binding region strikingly abolished the suppression of miR-497 on reporter gene expression (Figure 5b). RIP assays revealed that compared with the negative control, the enrichment level of MEF2C mRNA was substantially elevated by miR-497 overexpression (Figure 5c). In addition, qRT-PCR assays demonstrated that the expression of MEF2C mRNA was remarkably upregulated in the TAC model and Ang II-treated cardiomyocytes (Figure 5d and e). Moreover, in contrast to their counterparts, MEF2C protein expression was significantly decreased by miR-497 overexpression, and it was highly increased with in-miR-497 transfection (Figure 5f). We further determined whether TUG1 modulated the expression of MEF2C in cardiomyocytes. As expected, the MEF2C level was prominently elevated by TUG1 overexpression, and this effect was strongly abolished by miR-497 mimic transfection (Figure 5g).

### 3.6 MEF2C was a functional target of miR-497 in regulating Ang II-induced cardiomyocyte hypertrophy

**Figure 6:**
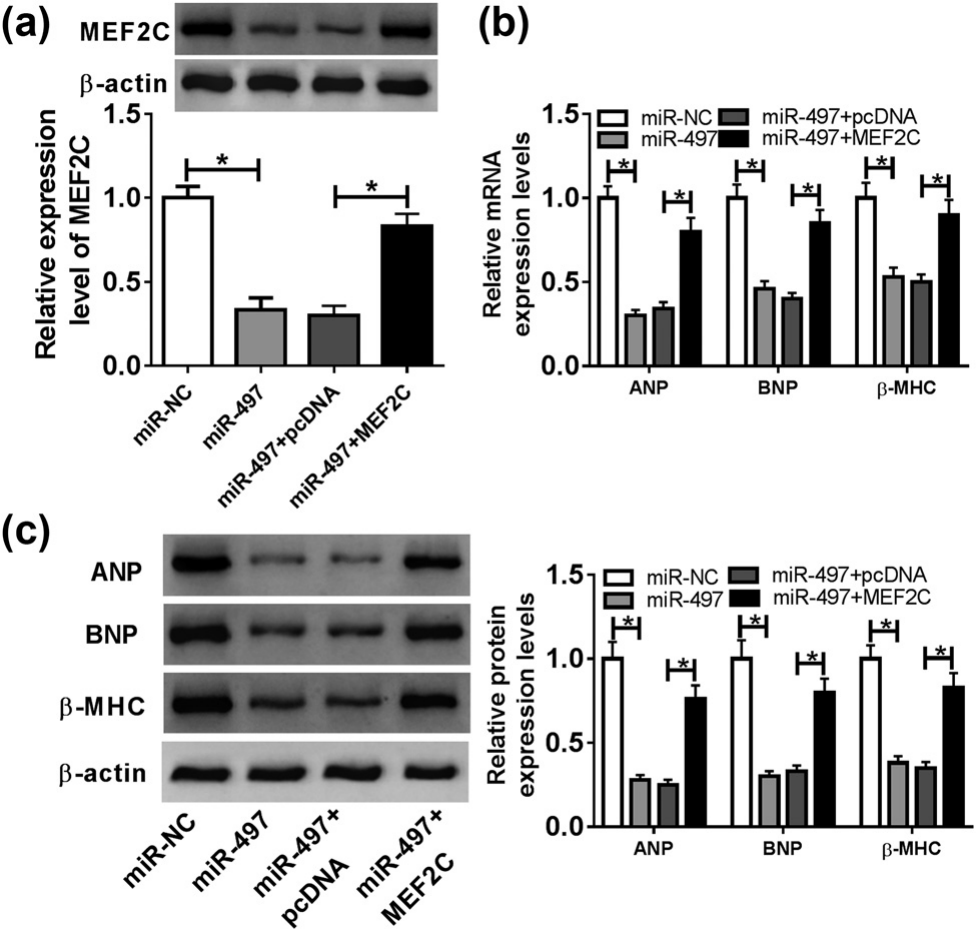
Overexpression of miR-497 exerted its antihypertrophic effect in Ang II-treated cardiomyocytes by MEF2C. Cardiomyocytes were transfected with miR-NC mimic, miR-497 mimic, miR-497 mimic + pcDNA-NC or miR-497 mimic + pcDNA-MEF2C before Ang II exposure, followed by the measurement of MEF2C protein expression by western blot (a), the mRNA levels of ANP, BNP and β-MHC by qRT-PCR (b) and their protein levels by western blot (c). *P < 0.05.

To provide further mechanistic insight into the link between miR-497 and MEF2C in cardiac hypertrophy, we cotransfected miR-497 mimic and pcDNA-MEF2C into cardiomyocytes before Ang II induction. In contrast to the negative control, the cotransfection of pcDNA-MEF2C significantly reversed the repressive effect of miR-497 overexpression on MEF2C expression (Figure 6a). Moreover, the restored level of MEF2C remarkably abolished the reduction of miR-497 overexpression on ANP, BNP and β-MHC levels in Ang II-treated cardiomyocytes (Figure 6b and c).

## 4 Discussion

Pathological cardiac hypertrophy has been widely known to cause the deposition of extracellular collagen, the loss of adrenergic responsivity and metabolic disorder, thereby leading to heart failure [19]. It was reported that cardiac hypertrophy could be established by Ang II induction *in vitro* and TAC surgery *in vivo* [17,20]. In the present study, we successfully established cardiac hypertrophy *in vivo* and *in vitro*, as evidenced by changes of hemodynamic parameters, the radio of heart weight and body weight and levels of ANP, BNP and β-MHC. Up to now, many lncRNAs have been identified as positive or negative regulators in cardiac hypertrophic pathways [21,22,23]. In this study, we first demonstrated that TUG1 alleviated cardiomyocyte hypertrophy in Ang II-induced cardiomyocytes through targeting the miR-497/MEF2C axis.

It was reported that TUG1 enhanced cardiac fibroblast transformation to myofibroblast by sponging miR-29c under hypoxia [24]. TUG1 had been found to play a crucial role in hypoxia-induced myocardial cell damage via regulating B-cell lymphoma 2 interacting protein 3 (Binp3) by sponging miR-145-5p [25]. In the current study, our data demonstrated that TUG1 was upregulated in the TAC rat model and Ang II-induced cardiomyocytes, and TUG1 knockdown attenuated Ang II-induced cardiomyocyte hypertrophy in agreement with the previous study [10].

Then, we used online database LncBase Predicted v.2 to help identify miRNAs that potentially bind to TUG1. Among these candidates, we selected miR-497 for further investigation, and we first verified that TUG1 acted as a miR-497 sponge. miR-497, a member of the miR-15 family, has been identified as a tumor-suppressive miRNA in a variety of human cancers, such as breast cancer [26], non-small cell lung cancer [27] and osteosarcoma [28]. Moreover, miR-497 was uncovered to be involved in the postnatal quiescence of skeletal muscle stem cells and osteoblast differentiation by targeting BMP signaling [29,30]. A previous document reported that miR-497 acted as a crucial regulator in cardiomyocyte mitotic arrest [31]. In the present study, our data revealed that miR-497 expression was downregulated in the TAC rat model and Ang II-induced cardiomyocytes, in accordance with a recent study [14]. Similar with the findings by Xiao et al. [14], we demonstrated that the enforced level of miR-497 rescued cardiomyocyte hypertrophy *in vitro*. Moreover, we first uncovered that miR-497 was a functional mediator of TUG1 in regulating Ang II-induced cardiomyocyte hypertrophy.

miRNAs are widely accepted to direct posttranscriptional repression of mRNA targets in cellular pathophysiology processes. Therefore, microT-CDS software was used to predict the potential targets of miR-497. Among these candidates, MEF2C was interesting in the present study owing to the crucial involvement of MEF2C in heart development, cardiomyocyte reprogramming and hypertrophic cardiomyopathy [32,33,34]. MEF2C has been emphasized as a signal-responsive mediator of the cardiac transcriptional program and plays a key role in the development of cardiac hypertrophy [35,36]. In addition, MEF2C deficiency alleviated the left ventricular hypertrophy induced by pressure overload through regulating the mTOR/S6K pathway in mice [37]. A previous study identified that MEF2C activation triggered by insulin-like growth factor-1 (IFG-1) mediated the pro-hypertrophic function of IGF-1 on the expression of cardiac gene [38]. In addition, the calcineurin-MEF2C pathway was demonstrated to participate in cardiac hypertrophy induced by endoplasmic reticulum stress in neonatal rat cardiomyocytes [39]. In the present study, we first verified that MEF2C was directly targeted and repressed by miR-497 in cardiomyocytes. Results of this study indicated that MEF2C was a functionally important target of miR-497 in modulating Ang II-induced cardiomyocyte hypertrophy. Similarly, Sato et al. reported that miR-214-3p played a repressive role in cardiac hypertrophy induced by Ang II via targeting MEF2C [29]. Xiao et al. demonstrated that the high level of miR-497 suppressed myocardial hypertrophy *in vitro* and *in vivo* through targeting Sirt4 [14]. More interestingly, we were first to validate that TUG1 modulated MEF2C expression through sponging miR-497. In addition, Zhao et al. highlighted that the silencing of TUG1 ameliorated diabetic cardiomyopathy-induced diastolic dysfunction via directly targeting miR-499-5p [40].

## 5 Conclusion

Our present study suggested that TUG1 knockdown alleviated Ang II-induced hypertrophy in cardiomyocytes at least in part through targeting the miR-497/MEF2C axis. The clinical significance of TUG1 and its potential value as a therapeutic target should be further explored.
